# Gastroenterology

**Published:** 1992-03

**Authors:** E. Rhys Davies


### GASTROENTEROLOGY
**Clinician's Guide to Nuclear Medicine**
**Harding L. K. and Robinson P. J. A.**
Churchill Livingstone, London, 1991, 140 pp.
ISBN 0 443 04418 X

This book is one of a series of handbooks commissioned by the British Nuclear Medicine Society to act as a guide to Clinicians interested in the clinical use of radioactive tracers. The book is designed to discuss the potential applications of radioactive trace methods to clinical problem solving.

No one would dispute the attraction of a series of this kind and after the choice of topic, the most important choice is that of the authors. This combination of Physician and Clinical Radiologist has the potential for success, because both of them have made a distinguished contribution to the development and clinical application of their speciality. The book is divided into a series of chapters which essentially describe the functional considerations that are the basis of the tests applied to a series of clinical problems. The choice of illustrations is judicious and effective. Each chapter has a small list of essential references at the end. The authors have succeeded in covering all the topics that are likely to be useful knowledge for both Registrars and Consultants and will introduce the reader deftly to most of the controversies surrounding the use of these tests. A little more might have been made of the use of splenic scanning in trauma and the only perplexing part of the book was the interpretation of Figure 11.3. Otherwise the book is written in the common sense and concise-style characteristic of these authors.

It is clear that the book has achieved the aims of the Editor in Chief, and presumably has fulfilled the aspirations of the authors. At a time when imaging textbooks are being published at a great rate and at great expense, it is important to ask whether there is a need for such a volume. The system based approach, the clarity of writing, the up-to-date discussion of clinical problems and the contrast with heavier tomes in the same general field make the answer unequivocally "yes".

E. Rhys Davies
3 September, 1991

